# A Physically-Modified Saline Suppresses Neuronal Apoptosis, Attenuates Tau Phosphorylation and Protects Memory in an Animal Model of Alzheimer's Disease

**DOI:** 10.1371/journal.pone.0103606

**Published:** 2014-08-04

**Authors:** Khushbu K. Modi, Arundhati Jana, Supurna Ghosh, Richard Watson, Kalipada Pahan

**Affiliations:** 1 Department of Neurological Sciences, Rush University Medical Center, Chicago, Illinois, United States of America; 2 Revalesio Corporation, Tacoma, Washington, United States of America; School of Medicine and Health Sciences, University of North Dakota, United States of America

## Abstract

Alzheimer's disease (AD), the leading cause of dementia in the aging population, is characterized by the presence of neuritic plaques, neurofibrillary tangles and extensive neuronal apoptosis. Neuritic plaques are mainly composed of aggregates of amyloid-β (Aβ) protein while neurofibrillary tangles are composed of the hyperphosphorylated tau protein. Despite intense investigations, no effective therapy is currently available to halt the progression of this disease. Here, we have undertaken a novel approach to attenuate apoptosis and tau phosphorylation in cultured neuronal cells and in a transgenic animal model of AD. RNS60 is a 0.9% saline solution containing oxygenated nanobubbles that is generated by subjecting normal saline to Taylor-Couette-Poiseuille (TCP) flow under elevated oxygen pressure. In our experiments, fibrillar Aβ1-42, but not the reverse peptide Aβ42-1, induced apoptosis and cell death in human SHSY5Y neuronal cells. RNS60, but not NS (normal saline), RNS10.3 (TCP-modified saline without excess oxygen) or PNS60 (saline containing excess oxygen without TCP modification), attenuated Aβ(1–42)-induced cell death. RNS60 inhibited neuronal cell death via activation of the type 1A phosphatidylinositol-3 (PI-3) kinase – Akt – BAD pathway. Furthermore, RNS60 also decreased Aβ(1–42)-induced tau phosphorylation via (PI-3 kinase – Akt)-mediated inhibition of GSK-3β. Similarly, RNS60 treatment suppressed neuronal apoptosis, attenuated Tau phosphorylation, inhibited glial activation, and reduced the burden of Aβ in the hippocampus and protected memory and learning in 5XFAD transgenic mouse model of AD. Therefore, RNS60 may be a promising pharmaceutical candidate in halting or delaying the progression of AD.

## Introduction

Alzheimer's disease (AD) [1] is a neurodegenerative disorder, resulting in progressive neuronal death and memory loss. Neuropathologically, the disease is characterized by two hallmark lesions: neurofibrillary tangles and neuritic plaques. Neuritic plaques are composed of aggregates of Aβ protein, a 40–43 amino acid proteolytic fragment derived from the amyloid precursor protein that is over-expressed in AD while NFTs are composed of hyperphosphorylated microtubule- associated protein tau [1], [2]. In the etiology of idiopathic AD, the mechanisms associated with Aβ neurotoxicity are not fully understood but appear to involve hyperphosphorylation of tau protein and activation of a pro-apoptotic pathway. Multiple lines of evidence demonstrate that errors in the regulation of either tau or the amyloid precursor protein result in neuronal death and cognitive dysfunction in humans [3], [4], [5]. Furthermore, experiments in both cultured rodent hippocampal neurons and transgenic mice demonstrate that Aβ-mediated neuronal death, learning and memory impairments require tau [6], [7].

Numerous studies have shown that tau phosphorylation is highly regulated via protein kinases functioning in various signal transduction pathways [8], [9]. One of these kinases is the serine/threonine kinase glycogen synthase kinase-3β (Gsk3β) [10], [11]. GSK3β sits at the convergence of several signaling pathways critical for proper neuronal functioning. Several apoptotic stimuli including Aβ are involved in modulating GSK3β activity, and consequently regulating tau phosphorylation. A large body of evidence has shown that GSK-3β robustly phosphorylates a majority of sites on tau both *in vitro* and *in vivo* [10], [12], [13]. Transgenic mice over-expressing GSK-3β display hyperphosphorylated tau protein, disrupted microtubules, and apoptotic neurons [14]. In addition, an increase in GSK3β immunoreactivity is observed in neurons bearing early stages of neurofibrillary tangles, suggesting that GSK3β is involved in the formation of neurofibrillary tangles.

Currently, drugs that are approved for use in AD do not prevent or reverse the disease progression and are only modestly efficacious while some might have adverse effects. Therefore, novel drugs are needed that protect or ameliorate progressive neuronal apoptosis and cell death in AD without serious side effects over long term usage. RNS60 is a physically modified saline generated by subjecting normal saline to Taylor-Couette-Poiseuille (TCP) flow under high oxygen pressure. It contains charge-stabilized nanobubbles, but no active pharmaceutical ingredients, and has broad anti-inflammatory activities [15]. Here we report that RNS60 prevents fibrillar Aβ-induced apoptosis, cell death, and tau phosphorylation in neuronal cells via type IA PI3K-Akt-GSK3β-Bad pathway. Furthermore, RNS60 treatment reduced neuronal apoptosis, Tau phosphorylation, glial activation, and Aβ load *in vivo* in the hippocampus and protected memory and learning in 5XFAD transgenic mouse model of AD. These results establish a novel mode of action of RNS60 and open an option for treating patients with neurodegenerative disorders with this simple TCP-modified saline as primary or adjunct therapy.

## Materials and Methods

Animal maintaining and experiments were in accordance with National Institute of Health guidelines and were approved by the Institutional Animal Care and Use committee of the Rush University of Medical Center, Chicago, IL. Whenever needed, animals were anesthetized by ketamine/xylazine injectables.

### Reagents

Neurobasal medium and B27/B27-AO supplement were purchased from Invitrogen (Carlsbad, CA) and fetal bovine serum (FBS) was obtained from Atlas Biologicals (Fort Collins, CO). L-Glutamine, DMEM/F-12 50/50 1x, Hank's balanced salt solution (HBSS) and 0.05% trypsin were purchased from Mediatech (Washington, DC). Antibiotic-antimycotic and Akt inhibitor (Akt-i) were obtained from Sigma (St. Louis, MO). GSK3-β inhibitor, LY294002 and rabbit anti-mouse iNOS antibodies were acquired from Calbiochem (Gibbstown, NJ). Human Aβ peptides (1–42) and (42–1) were obtained from Bachem Bioscience. Rat anti-mouse Iba1 was purchased from Chemicon. Aβ (N) 82E1 monoclonal antibodies were received from IBL America (Minneapolis, MN). Alexa-fluor antibodies used in immunostaining were obtained from Jackson ImmunoResearch and IR-dye-labeled reagents used for immunoblotting were from Li-Cor Biosciences.

### Preparation of Fibrillar Aβ

**Figure 1:**
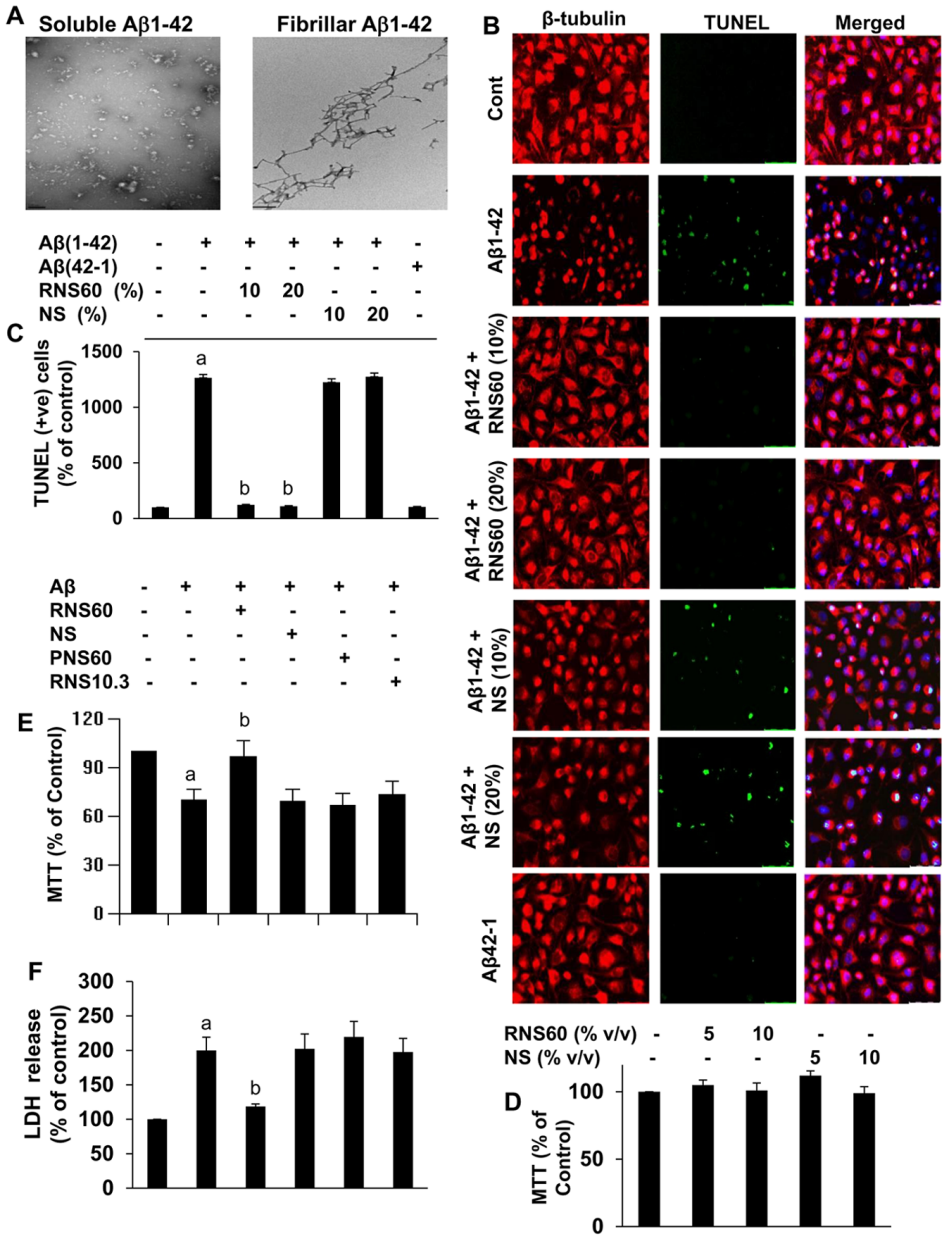
RNS60 strongly inhibits fibrillar (Aβ1–42)-induced apoptosis in SHSY5Y neuronal cells. *A*, Morphology of soluble and fibrillar form of (Aβ1–42) peptides was examined by transmission electron microscopy. *B*, SHSY5Y neuronal cells were either pretreated with different concentrations of RNS60 or NS for 1 h in Neurobasal medium containing 2% B27-AO followed by insult with 1 µM fibrillar Aβ1–42 for 6 h. Apoptotic events were detected by TUNEL. *C*, Digital images were collected under bright-field setting using a 20× objective. TUNEL-positive neurons were counted manually in four different images of each of three coverslips. Values obtained from the control group served as 100%, and data obtained in other groups were calculated as percent of control accordingly. Results are mean ± S.D. of three different experiments. ^a^p<0.001 vs control; ^b^p<0.001 vs Aβ1–42. D, Cells were treated with different concentrations of RNS60 and NS for 24 h followed by monitoring cell viability by MTT assay. Results are mean ± S.D. of three different experiments. Cells preincubated with 10% RNS60, NS, RNS10.3, and PNS60 for 1 h were stimulated by Aβ. After 24 h of stimulation, cell viability was monitored by MTT (E) and LDH release (F). Results are mean ± S.D. of three different experiments. ^a^p<0.01 vs control; ^b^p<0.01 vs Aβ1–42.

Fibrillar Aβ1–42 and control reverse peptide Aβ42–1 (Bachem Bioscience) were prepared by incubating freshly solubilized peptides at 50 µM in sterile distilled water at 37°C for 5 days [16]. See figure 1A for the morphology of fibrillar forms of Aβ1–42.

### Treatment of SHSY5Y human neuroblastoma cells

SHSY5Y cells were purchased from ATCC (Manassas, VA). These cells were cultured in DMEM/F-12 containing 10% FBS for 24 h followed by replacing the media by neurobasal medium containing 2% B27 supplement. After 48 h, media was replaced by neurobasal medium containing B27 supplement without antioxidant (B27-AO) before treatment with fibrillar Aβ peptides, RNS60/PNS60/RNS10.3/NS, and different signaling molecules.

### Isolation of cortical neurons

Fetal (E18-E16) mouse cortical neurons were prepared as previously described by us [17]. Briefly, whole brains were removed and cortices dissected in serum-free Neurobasal media. Cortical cells were then centrifuged, dissociated and plated in poly-D-lysine-coated 8-well chamber slides. After 5 min, the non-adherent cell suspension was discarded and Neurobasal media supplemented with 2% B27 was added to each well. Cells were incubated for 5 days followed by treatment with RNS60 and/or Aβ1–42 in Neurobasal media supplemented with 2% B27 minus antioxidants (Invitrogen).

### Preparation of RNS60

RNS60 was generated at Revalesio (Tacoma, WA) using Taylor-Couette-Poiseuille (TCP) flow as described before [15], [18], [19]. Briefly, sodium chloride (0.9%) for irrigation, USP pH 5.6 (4.5–7.0, Hospira), was processed at 4°C and a flow rate of 32 mL/s under 1 atm of oxygen back-pressure (7.8 mL/s gas flow rate), while maintaining a rotor speed of 3,450 rpm. Chemically, RNS60 contains water, sodium chloride, 50–60 parts/million oxygen, but no active pharmaceutical ingredients.

Following controls for RNS60 were also used in this study: a) NS, normal saline from the same manufacturing batch. This saline contacted the same device surfaces as RNS60 and was bottled in the same way and b) PNS60, saline with same oxygen content (55±5 ppm) that was prepared inside of the same device but was not processed with TCP flow. Careful analysis demonstrated that all three fluids were chemically identical [15]. Liquid chromatography quadrupole time-of-flight mass spectrometric analysis also showed no difference between RNS60 and other control solutions [15]. On the other hand, by using atomic force microscopy, we studied nanobubble nucleation in RNS60 and other saline solutions and observed that RNS60 has a nanobubble composition different from that of control saline solutions [15]. This same relative pattern of nanobubble number and size was observed when positive potentials were applied to AFM surfaces with the same control solutions, suggesting the involvement of charge in stabilization of nanobubbles in RNS60 [15].

### Animals

B6SJL-Tg(APPSwFlLon,PSEN1*M146L*L286V)6799Vas/J transgenic (5XFAD) mice were purchased from Jackson Laboratories (Bar Harbor, ME). Five month old male 5XFAD mice were treated with RNS60 or NS (300 µl/mouse/2d) via i.p. injection for 2 month followed by monitoring memory and learning and hippocampal histochemical and biochemical assays.

### Barnes Maze and T Maze

Maze experiments were performed as described by us [20], [21]. Briefly, for Barnes maze, mice were trained for 2 consecutive days followed by examination on day 3. During training, the overnight food-deprived mouse was placed in the middle of the maze in a 10 cm high cylindrical black start chamber. After 10 s, the start chamber was removed to allow the mouse to move around the maze to find out the color food chips in the baited tunnel. The session was ended when the mouse entered the baited tunnel. The tunnel was always located underneath the same hole (stable within the spatial environment), which was randomly determined for each mouse. After each training session, maze and escape tunnel were thoroughly cleaned with a mild detergent to avoid instinctive odor avoidance due to mouse's odor from the familiar object. On day 3, the maze was illuminated with high wattage light that generated enough light and heat to motivate animals to enter into the escape tunnel [22], allowing us to measure latency (duration before all four paws were on the floor of the escape box) and errors (incorrect responses before all four paws were on the floor of the escape box).

For T maze, mice were also habituated in the T-maze for two days under food-deprived conditions so that animals can eat food rewards at least five times during 10 minutes period of training. During each trial, mice were placed in the start point for 30 s and then forced to make a right arm turn which was always baited with color food chips. On entering the right arm, they were allowed to stay there for 30–45 s, then returned to the start point, held for 30 s and then allowed to make right turn again. As described above, after each training session, T maze was thoroughly cleaned with a mild detergent. On day 3, mice were tested for making positive turns and negative turns. The reward side is always associated with a visual cue. Number of times the animal eats the food reward would be considered as a positive turn.

### Novel Object Recognition Task

Novel object recognition task was performed to monitor the short term memory as described by others [23] and us [21]. Briefly, during training, mice were placed in a square novel box (20 inches long by 8 inches high) surrounded with infrared sensor. Two plastic toys (between 2.5 and 3 inches) that varied in color, shape, and texture were placed in specific locations in the environment 18 inches away from each other. The mice were able to explore freely the environment and objects for 15 min and then were placed back into their individual home cages. After 30 mins, mice were placed back into the environment with two objects in the same locations, but now one of the familiar objects was replaced with a third novel object. The mice were then again allowed to explore freely both objects for 15 min. The objects were thoroughly cleaned with a mild detergent.

### Immunoblotting

Western blotting was performed as described earlier [24], [25], [26] with modifications. Briefly, cells were scraped in lysis buffer, transferred to microfuge tubes and spun into pellet. The supernatant was collected and analyzed for protein concentration via the Bradford method (Bio-Rad). SDS sample buffer was added to 30–50 µg total protein and the sample was boiled for 5 min. Denatured samples were electrophoresed on NuPAGE Novex 4–12% Bis-Tris gels (Invitrogen) and proteins transferred onto a nitrocellulose membrane (Bio-Rad) using the Thermo-Pierce Fast Semi-Dry Blotter. The membrane was then washed for 15 min in TBS plus Tween 20 (TBST) and blocked for 1 h in TBST containing BSA. Next, membranes were incubated overnight at 4°C under shaking conditions with primary antibody. The next day, membranes were washed in TBST for 1 h, incubated with secondary antibody (Li-Cor Biosciences) for 1 h at room temperature, washed for one more hour and visualized under the Odyssey Infrared Imaging System (Li-COR, Lincoln, NE).

### Immunostaining

Coverslips containing 200–300 cells/mm^2^ were fixed with 4% paraformaldehyde for 20 min followed by treatment with cold ethanol (−20°C) for 5 min and 2 rinses in PBS. The samples were blocked with 3% bovine serum albumin in PBS containing Tween 20 (PBST) for 30 min and incubated in PBST containing 1% bovine serum albumin and goat anti-MAP-2 (1∶50), as described previously [25], [26], [27]. After three washes in PBST (15 min each), the slides were further incubated with Cy5 (Jackson ImmunoResearch Laboratories, Inc.). For negative controls, a set of culture slides was incubated under similar conditions without the primary antibodies. The samples were mounted and observed under an Olympus IX81 fluorescent microscope. For tissue staining, brains were kept in 4% paraformaldehyde and 30-µm slices were sectioned in a cryostat followed by immunostaining as described before [28], [29].

### Fragment End Labeling of DNA

Fragmented DNA was detected *in situ* by the terminal deoxynucleotidyltransferase-mediated binding of 3′-OH ends of DNA fragments generated in response to fibrillar Aβ1–42, using a commercially available kit (TdT FragE, Calbiochem) as described before [27]. Briefly, cover slips were treated with 20 µg/ml proteinase K for 15 min at room temperature and washed prior to terminal deoxynucleotidyltransferase staining.

### Cell Viability Measurement

#### MTT assay

Mitochondrial activity was measured with the 3-(4, 5-dimethylthiazol-2-yl)-2, 5-diphenyltetrazolium bromide (MTT) assay (Sigma). The cells were grown on 24-well culture plates with 500 µl of medium and treated with various reagents according to the experimental design. At the end of the treatment period, 300 µl of culture medium were removed from each well, and 20 µl of MTT solution (5 mg/ml) were added and incubated for 1 h.

### Lactate Dehydrogenase Measurement

The activity of lactate dehydrogenase (LDH) was measured using the direct spectrophotometric assay using an assay kit from Sigma.

### Transmission electron microscopy (TEM) sample preparation

Aliquots of sample (5 µl) were added onto the surface of carbon-coated electron microscope grids and adsorbed for 2 min at room temperature. After rinsing with 20 µl of sterile deionized water, 5 µl of 1% (w/v) uranyl acetate was added for 15–20 s. Grids were then blotted dry and examined under a JEOL JEM-1220 transmission microscope.

### Statistical Analysis

All values were expressed as means ± SD of three independent experiments. Statistical differences between means were calculated by the Student's *t*-test. A *p*-value of less than 0.05 (p<0.05) was considered statistically significant. Differences in behavioral measures were examined by independent one-way ANOVA using SPSS. Homogeneity of variance between test groups was examined using Levene's test. *Post-hoc* analyses were conducted using Tukey's or Games-Howell tests, where appropriate. *p*<0.05 was considered statistically significant.

## Results

### RNS60 protects human SHSY5Y neuronal cells against Aβ toxicity

**Figure 2:**
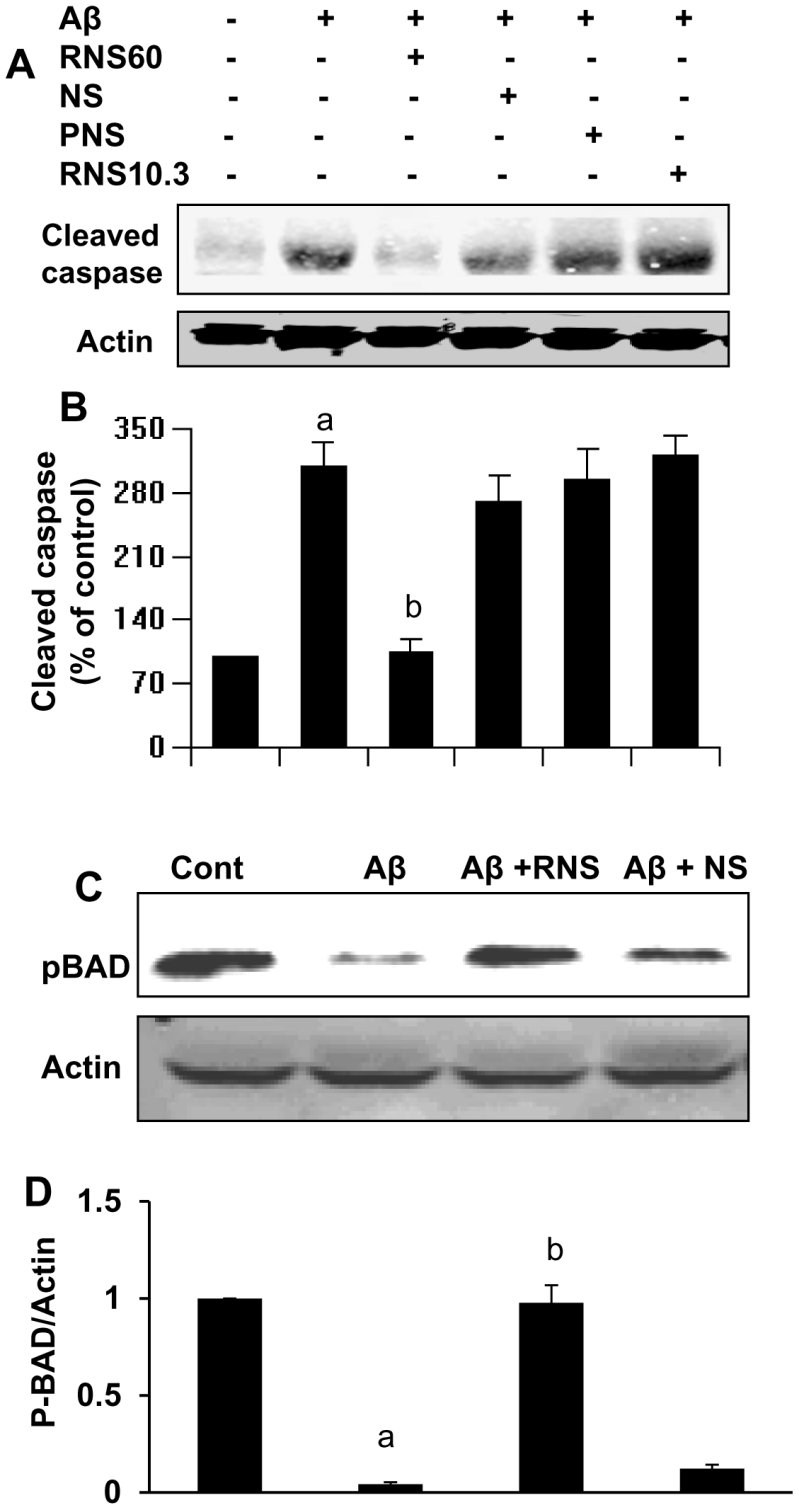
RNS60 suppresses fibrillar (Aβ1–42)-induced apoptotic signaling pathway in SHSY5Y neuronal cells. *A*, Cells preincubated with 10% RNS60, NS, RNS10.3, and PNS60 for 1 h were stimulated by Aβ. After 4 h of stimulation, cells lysates were analyzed for cleaved caspase by Western blot. Membranes were stripped and reprobed with anti-β-actin antibody. *B*, Bands were scanned and results presented as protein expression relative to Actin. Results are expressed as mean ± SD of three different experiments. *^a^p<0.001 vs control; ^b^p<0.001 vs Aβ1-42*. *C*, Cells were pretreated with 10% v/v RNS60 or NS for 1 h in followed by exposure to Aβ (1 µM). After 3 h of challenge, cell lysates were prepared and analyzed by Western blotting with antibodies against phospho-BAD. Membranes were stripped and reprobed with anti-β-actin antibody. *D*, Bands were scanned and results presented as protein expression relative to Actin. Results are expressed as mean ± SD of three independent experiments. *^a^p<0.001 vs control; ^b^p<0.001 vs Aβ1-42*.

Because the fibrillar form of Aβ is commonly found in the senile plaques in AD brains [1] and is known to cause neuronal death, we first examined whether fibrillar Aβ1–42 was capable of inducing apoptosis in the SHSY5Y cell line in our experimental setting. As demonstrated in figure 1B, fibrillar Aβ1–42 peptide, but not the reverse Aβ42–1 peptide, markedly induced the formation of apoptotic bodies after 6 h of stimulation as seen by TUNEL staining. Pretreatment of SHSY5Y cells for 1 h with RNS60 inhibited Aβ- induced apoptosis in a dose dependent manner, while pretreatment with different doses of NS did not show any protective effect in Aβ-treated cells (Fig. 1B–C). We also examined the protective effect of RNS60 against Aβ-induced neurotoxicity using the MTT and LDH release assays. RNS60 and NS alone did not alter the MTT metabolism (Fig. 1D), suggesting that these saline solutions were not toxic to neuronal cells. Aβ significantly decreased the viability of SHSY5Y cells as monitored by a decrease in MTT metabolism (Fig. 1E) and an increase in LDH release (Fig. 1F). RNS60 effectively reduced the Aβ-induced loss of MTT metabolism as well as the increased LDH (Fig. 1E–F). In contrast, NS, PNS60 (saline containing excess oxygen in the absence of TCP modification) or RNS10.3 (TCP-modified saline without excess oxygen) failed to rescue the Aβ-induced loss of cell viability (Fig. 1E–F). It is known that caspase-3 activation plays a crucial role in apoptosis. As evident from figure 2A–B, RNS60 treatment caused a decrease in the proteolytically active form of caspase-3 after 4 h of Aβ stimulation while NS, PNS60 and RNS10.3 were ineffective.

Next, we investigated the neuroprotective role of RNS60 in response to Aβ toxicity from another angle. Bad is a pro-apoptotic member of the Bcl-2 family that promotes cell death by binding to Bcl-2, an anti-apoptotic member, and inhibiting its function [30]. The apoptotic activity of Bad is inhibited by activation of intracellular signaling pathways that result in the phosphorylation of Bad at Ser112 and Ser136. Phosphorylation at these sites [31], [32] promotes binding of Bad to 14-3-3 proteins, thereby sequestering it in the cytosol and preventing its association with Bcl-2 [30]. As shown in figure 2C–D, Aβ-induced dephosphorylation of BAD is inhibited by RNS60, but not by NS. Together, these findings suggest a neuroprotective effect of RNS60 in Aβ-induced neurotoxicity.

### RNS60 treatment attenuates neuronal apoptosis in vivo in the hippocampus of 5XFAD mice

**Figure 3:**
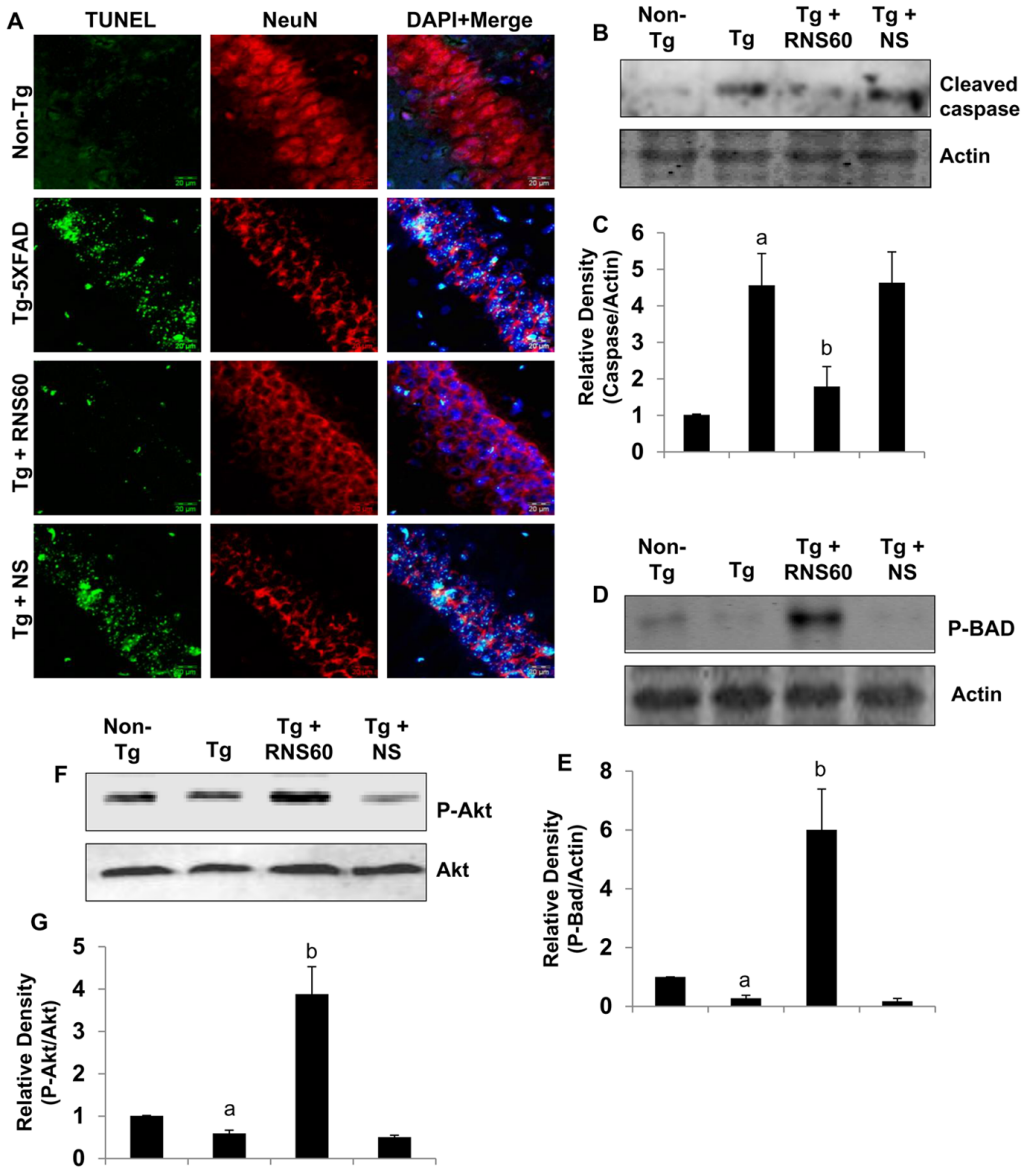
RNS60 treatment inhibits neuronal apoptosis *in vivo* in the hippocampus of Tg5XFAD mice. Tg mice (5 months old) were treated with RNS60 and NS (300 µl/mouse/2d) via i.p. injection and after 2 months of treatment, hippocampal sections were double-labeled for TUNEL and NeuN (A). Results represent analysis of two hippocampal sections of each of five mice per group. Tissue lysates were analyzed for cleaved caspase 3 (B&C), phospho-BAD (D&E) and phospho-Akt/total Akt (F&G) by Western blot. Bands were scanned and results presented as relative density (C, E & G). Results represent mean ± SEM of four mice per group. *^a^p<0.001 vs non-Tg; ^b^p<0.001 vs Tg*.

Neuronal apoptosis is often observed in AD brain, and its reversal may have beneficial effects in AD. Therefore, we tested the effect of RNS60 treatment on neuronal apoptosis in the hippocampus of 5XFAD mice, an accelerated model of AD. Five month old male 5XFAD mice were treated with RNS60 or NS (300 µl/mouse/2d) via i.p. injection for 2 months. After 2 months of treatment, neuronal apoptosis was detected by double-labeling of hippocampal sections for NeuN and TUNEL by immunostaining and Western blot analysis. As expected, a number of TUNEL-positive bodies co-localized with NeuN in the CA1 region of the hippocampus of 5XFAD mice as compared to age-matched non-transgenic (non-Tg) mice (Fig. 3A). Treatment of 5XFAD mice with RNS60 led to marked suppression of neuronal apoptosis in the hippocampus (Fig. 3A). This result was confirmed by detection of several other molecules. As shown in figure 3B–C, RNS60 treatment reduced the elevated level of cleaved caspase 3 in the hippocampus of 5XFAD mice (Fig. 3B–C). Since Aβ-induced dephosphorylation of BAD was inhibited by RNS60 in SHSY5Y cells (Fig. 2C–D), we examined the status of phospho-Bad in the hippocampus of RNS60-treated and untreated 5XFAD mice. As expected, the level of phospho-Bad decreased in the hippocampus of 5XFAD mice (Fig. 3D–E). However, treatment of 5XFAD mice with RNS60 led to significant increase in phospho-Bad in the hippocampus of 5XFAD mice (Fig. 3D–E). In addition, RNS60 treatment augmented the level of phospho-Akt, which is decreased in the hippocampus of 5XFAD mice as compared to non-Tg mice (Fig 3F–G), suggesting that consistent with the in vitro dataRNS60 treatment is capable of upregulating anti-apoptotic signaling pathways *in vivo* in the hippocampus of 5XFAD mice in a similar manner to that found in neuronal cells in vitro.

### RNS60 activates the PI3K - Akt pathway

**Figure 4:**
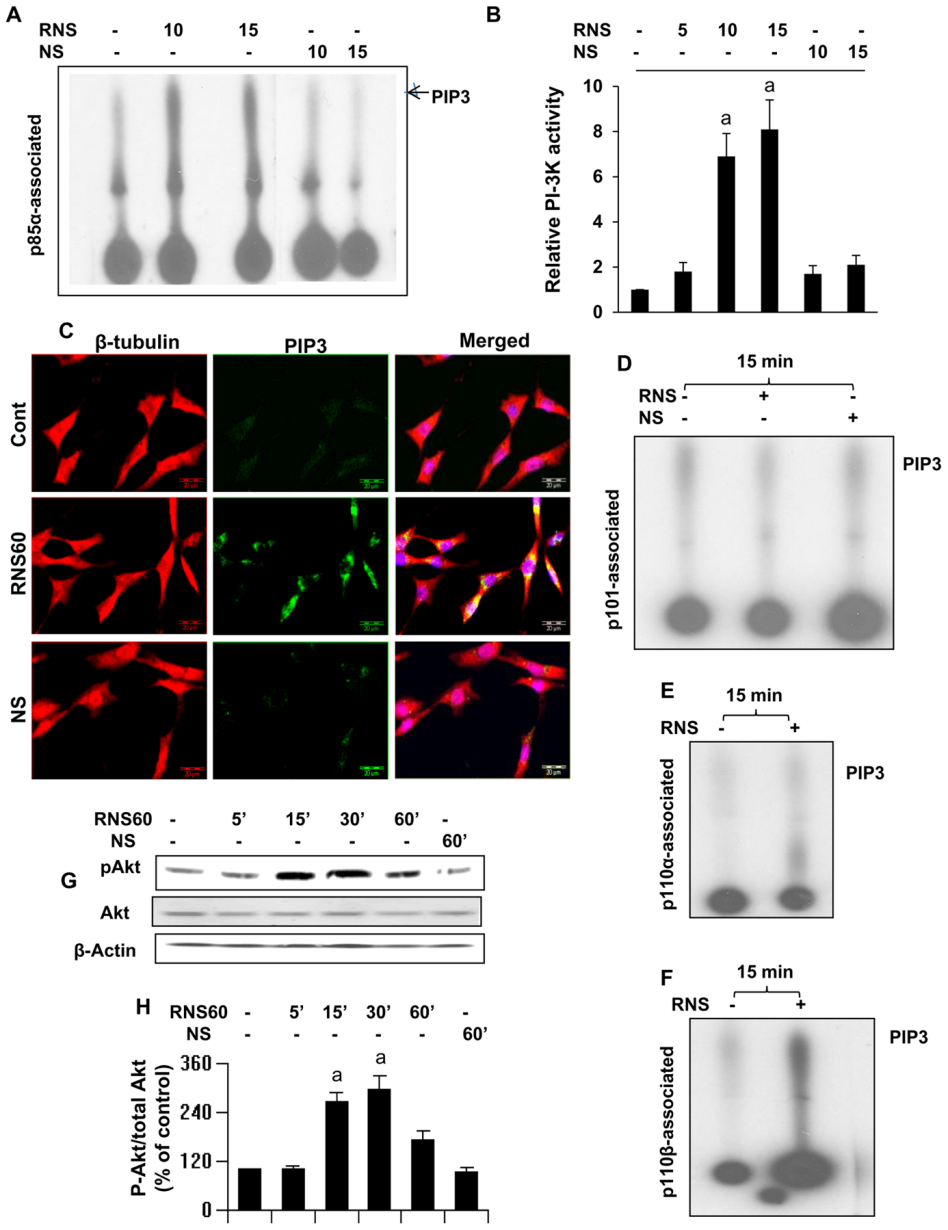
Activation of PI3K by RNS60 in SHSY5Y neuronal cells. Cells were treated with either 10% RNS60 or NS in B27-AO supplemented media. At different time points, cells were lysed, immunoprecipitated with antibodies against either p85α (*A*), p101 (*D*), p110α (*E*), or p110 β (*F*), and the lipid kinase activity of immunoprecipitated PI3K was assayed as described in *Materials and Methods*. Lipids were detected by exposure of film at −70°C (*A, D–F*) and quantified by densitometry (*B*). Results represent mean ± SD of three different experiments. *^a^p<0.001 vs control*. *C*, Cells were treated with RNS60 or NS for 15 min and the level of PIP3 was monitored by immunofluorescence. Cells were incubated either with 10% RNS60 or NS for the indicated periods of time. After stimulation, cells lysates were prepared and analyzed by Western blotting with antibodies specific for anti-phospho-Akt or total Akt. Blots were stripped and reprobed with anti-β-actin antibody. *B*, Bands were scanned and results presented as ratio of phospho-Akt to total Akt. Results represent mean ± SD of three different experiments. *^a^p<0.001 vs control*.

Next, we investigated mechanisms underlying the anti-apoptotic effects of RNS60. The PI3K pro-survival pathway plays an important role in mechanisms of neuroprotection. Recently we have observed that RNS60 activates class IA PI3K in microglial cells [15]. Therefore, we tested the effect of RNS60 on PI3K activation in neuronal cells. Class IA PI3K, which is regulated by receptor tyrosine kinases, consists of a heterodimer of a regulatory 85-kDa subunit and a catalytic 110-kDa subunit (p85:p110α/β/δ). Class IB PI3K, on the other hand, consists of a dimer of a 101-kDa regulatory subunit and a p110γ catalytic subunit (p101/p110γ). As evident from figure 4A–B, RNS60 markedly induced the activation of p85α-associated PI3K within 10–15 minutes of stimulation, suggesting the activation of class IA PI3K by RNS60. Phosphatidylinositol (3,4,5)-triphosphate (PIP_3_) is the product of class I PI 3-kinase. Therefore, to confirm the activation of class I PI 3-kinaseby monitoring PIP_3_ levels in RNS60-treated SHSY5Y neuronal cells by immunofluorescence. RNS60 increased the level of PIP_3_ at 15 minutes of stimulation (Fig. 4C). RNS60 did not, however, activate PI 3-kinase activity associated with p101 (Fig. 4D), suggesting that it is unable to activate class IB PI 3-kinase in neuronal cells. Using an immuno-complex lipid kinase assay, we could demonstrate that RNS60 induced activation of PI 3-kinase through p110β (Fig. 4E), but not p110α (Fig. 4F).

PI-3 kinases affect diverse cellular functions, many of which are linked to activation of the protein kinase Akt. Therefore, we examined whether RNS60 activates Akt in SHSY5Y neuronal cells. RNS60, significantly induced the activation of Akt in neuronal cells as shown by immunoblot analysis of phosphorylated Akt, without increasing the overall level of total Akt protein (Fig. 4G–H). Exposing the cells to 2 µM LY294002 (PI3K inhibitor) and 2 µM Akti (Akt inhibitor) abrogated the protective effect of RNS60 on Aβ-mediated death of neuronal cells (data not shown), suggesting that activation of PI3K-Akt signaling is essential for the neuroprotective effect of RNS60 against Aβ toxicity.

### RNS60 attenuates Aβ-induced neuronal apoptosis via the PI3K – Akt pathway

**Figure 5:**
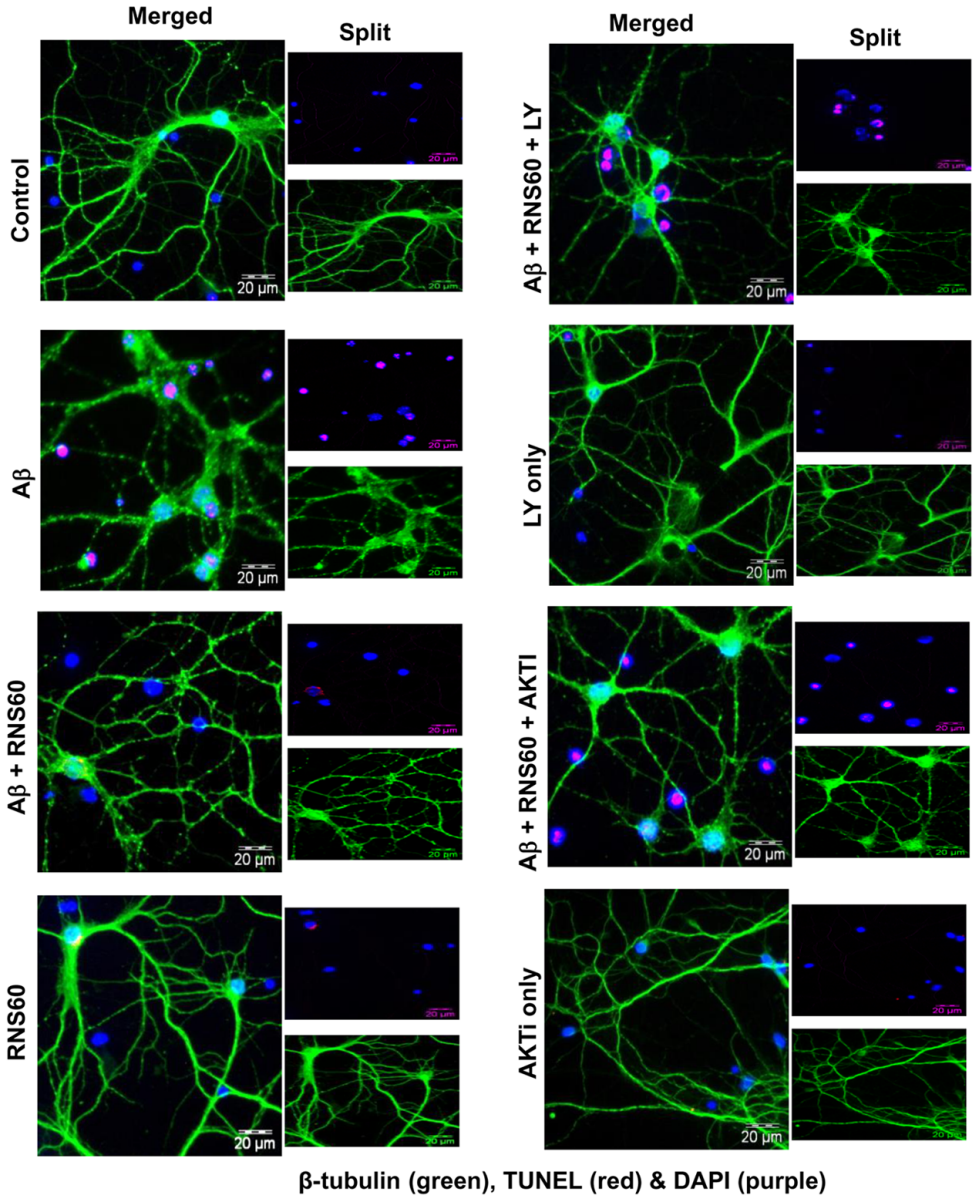
RNS60 attenuates neuronal apoptosis via PI3K – Akt pathway. Mouse primary cortical neurons preincubated with 2 µM LY294002 (PI3K inhibitor) and 2 µM Akti (Akt inhibitor) for 15 min were treated with 5% v/v RNS60 for 30 min followed by challenge with 1 µM fibrillar Aβ1–42. After 6 h, cells were double-labeled for β-tubulin and TUNEL. Results represent three different experiments.

Since RNS60 suppressed Aβ-induced apoptosis in SHSY5Y neuronal cells, we examined whether RNS60 was capable of doing so in primary neurons. As expected, fibrillar Aβ1–42 peptides induced apoptosis in primary cortical neurons (Fig. 5). However, pretreatment of cortical neurons with RNS60, but not NS, inhibited Aβ-induced apoptosis (Fig. 5), suggesting that RNS60 is also capable of inhibiting apoptosis in primary neurons. Since RNS60 induced the activation of PI3K and Akt (Fig. 4), we examined whether RNS60 employed this pathway to suppress apoptosis in Aβ-insulted neurons. Abrogation of RNS60-mediated protection of cortical neurons by LY294002 (PI3K inhibitor) and Akti (Akt inhibitor) (Fig. 5) suggests that activation of PI3K-Akt signaling is essential for the neuroprotective effect of RNS60 against Aβ toxicity.

### RNS60 inhibits Aβ-induced tau phosphorylation in SHSY5Y neuronal cells

**Figure 6:**
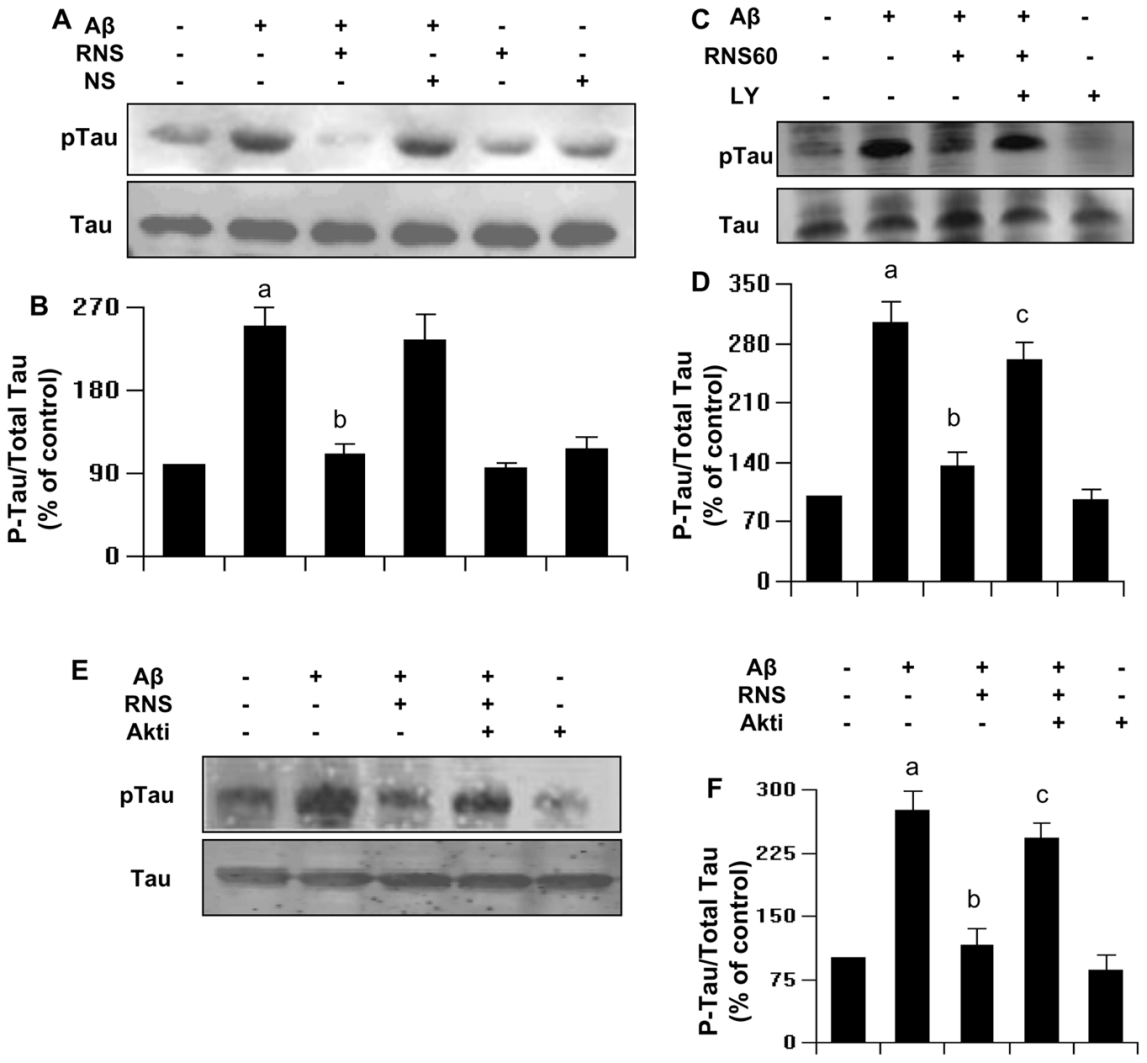
RNS60 inhibits Aβ-induced phosphorylation of Tau in SHSY5Y neuronal cells via PI3K – Akt pathway. *A*, Cells preincubated with 10% RNS60 and NS for 1 h were stimulated by Aβ. After 3 h of challenge, cell lysates were analyzed by Western blotting with antibodies against phospho-Tau and Tau. *B*, Bands were scanned and results presented as protein expression relative to Tau. Results are expressed as mean ± SD of three independent experiments. *^a^p<0.001 vs control; ^b^p<0.001 vs Aβ*. *C*, RNS60-mediated prevention of Aβ-induced tau phosphorylation was also blocked by LY in SHSY5Y cells as monitored by immunoblotting under similar experimental set up. *D*, Phosphorylated Tau was quantified as densitometry values, which were normalized to Tau. Results are mean ± S.D. of three different experiments. *^a^p<0.001 vs Control; ^b^p<0.001 vs Aβ; ^c^p<0.001 vs (Aβ+RNS60)*. *E*, RNS60-mediated prevention of Aβ-induced tau phosphorylation was also blocked by Akti in SHSY5Y cells. *F*, Phosphorylated Tau was quantified as densitometry values, which were normalized to Tau. Results are mean ± S.D. of three different experiments. *^a^p<0.001 vs Control; ^b^p<0.001 vs Aβ; ^c^p<0.001 vs (Aβ+RNS60)*.

It has been reported that Aβ-induced tau phosphorylation in neurons is involved in neuronal death [33]. Therefore, in order to evaluate whether the neuroprotective effect of RNS60 is also related to tau phosphorylation, we examined the levels of phospho-tau in SHSY5Y cells treated with 1 µM of Aβ. As seen by Western blot analysis of phospho-tau (Fig. 6A–B), Aβ significantly increased phosphorylation levels of tau protein after 3 h of stimulation. Pretreatment of neuronal cells with RNS60 effectively inhibited tau phosphorylation (Fig. 6A–B).

### RNS60 treatment attenuates tau phosphorylation in the hippocampus of 5XFAD mice

**Figure 7:**
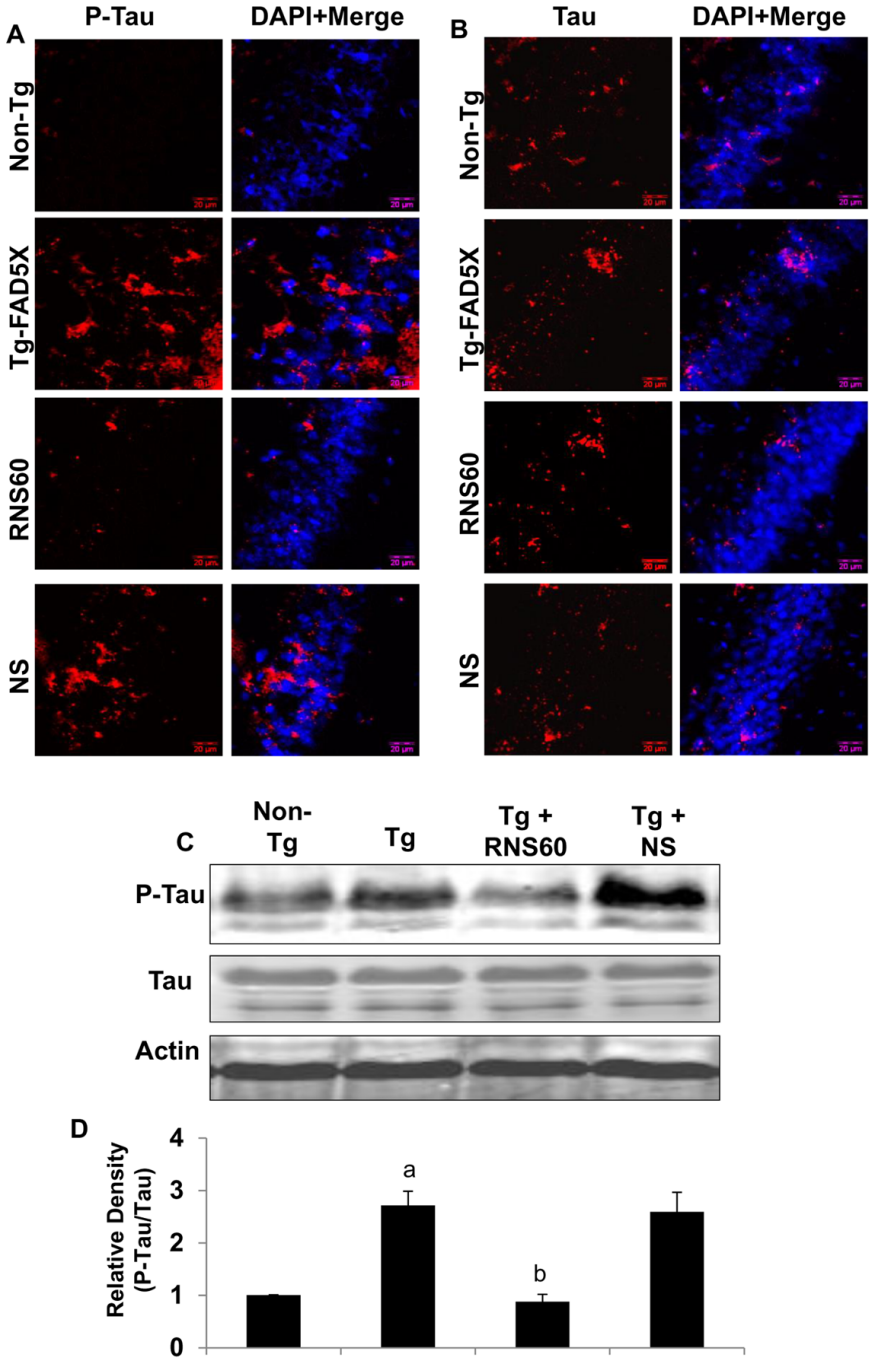
RNS60 treatment attenuates phosphorylation of tau in the hippocampus of Tg 5XFAD mice. Tg mice (5 months old) were treated with RNS60 and NS (300 µl/mouse/2d) via i.p. injection and after 2 months of treatment, phospho-tau (A) and total tau (B) were monitored in the hippocampus by immunofluorescence. Results represent analysis of two hippocampal sections of each of five mice per group. C, Tissue lysates were analyzed for phospho-tau and total tau by Western blot. *D*, Bands were scanned and results presented as ratio of phospho-tau to total tau. Results represent mean ± SEM of four mice per group. *^a^p<0.001 vs non-Tg; ^b^p<0.001 vs Tg*.

Since RNS60 inhibited tau phosphorylation in SHSY5Y cells, we examined the effect of RNS60 treatment on the status of tau phosphorylation *in vivo* in the hippocampus of 5XFAD mice. Immunofluorescence analysis indicates a marked increase in phospho-tau in hippocampal sections of 5XFAD mice as compared to non-Tg mice (Fig. 7A). Similar to our findings in cultured cells, RNS60 suppressed the level of phospho-tau in the hippocampus without affecting the total level of tau protein, as shown both by immunofluorescence (Fig. 7A–B) and by Western blot analysis (Fig. 7C–D). These results demonstrate that RNS60 treatment is capable of decreasing tau phosphorylation *in vivo* in the hippocampus of 5XFAD mice.

### How does RNS60 inhibit Aβ-induced tau phosphorylation?

Since RNS60 induces activation of PI3K–Akt pathway, we examined whether RNS60 requires this pathway to suppress tau phosphorylation. SHSY5Y neuronal cells were pretreated in the presence or absence of the PI3K inhibitor LY294002 and RNS60, followed by stimulation with Aβ. LY294002 negated the RNS60-mediated down-regulation of phospho-tau in Aβ treated neuronal cells (Fig. 6C–D). Similarly, the Akt inhibitor Akti also blocked RNS60-mediated suppression of tau phosphorylation in neuronal cells in culture (Fig. 6E–F). These findings demonstrate that RNS60 suppresses tau phosphorylation in neurons via the PI3K-Akt pathway.

### How does the PI3K-Akt pathway couple RNS60 to the suppression of tau phosphorylation?

**Figure 8:**
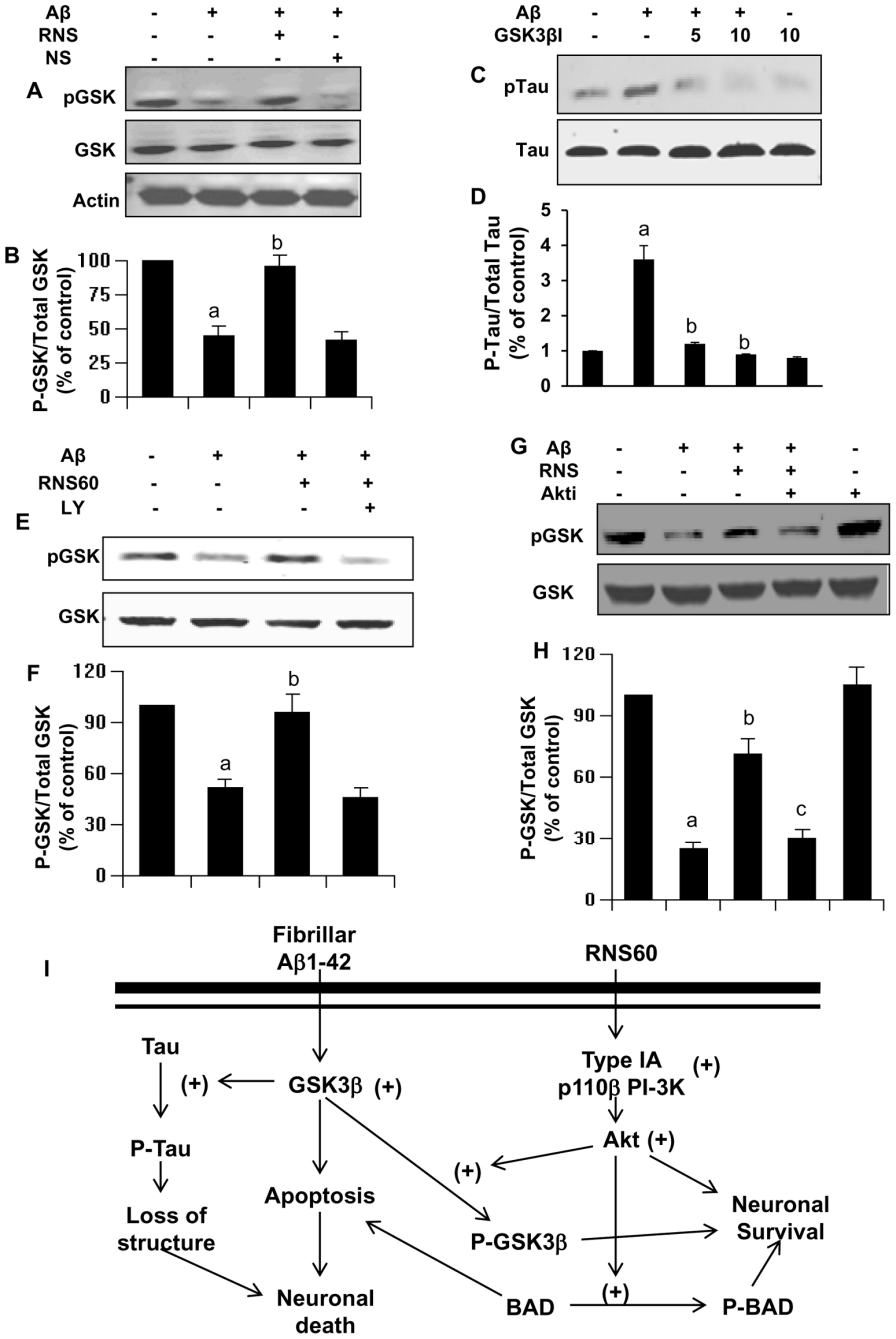
Aβ1–42 insult increased the phosphorylation of tau via activation GSK and RNS60 suppressed GSK activation through PI3K – Akt pathway in SHSY5Y neuronal cells. *A*, Cells preincubated with 10% RNS60 and NS for 1 h were stimulated by Aβ followed by monitoring the levels of phospho-GSK3β by Western blot. *B*, Bands were scanned and results presented as phospho-GSK/total GSK. Results are expressed as mean ± SD of three independent experiments. *^a^p<0.001 vs control; ^b^p<0.001 vs Aβ*. *C*, Cells were incubated with or without GSK-3βI for 30 min followed by Aβ stimulation for 3 h. Tau phosphorylation was measured by Western blot. *D*, Phosphorylated Tau was quantified as densitometry values normalized to Tau. Results are expressed as mean ± SD of three independent experiments. *^a^p<0.001 vs Control; ^b^p<0.001 vs Aβ*. Cells pretreated with RNS60 or RNS60 plus LY for 30 min were challenged with Aβ followed by monitoring the phosphorylation of GSK by Western blot. Bands were scanned and results presented as phospho-GSK/total GSK. Results are expressed as mean ± SD of three independent experiments. *^a^p<0.001 vs control; ^b^p<0.001 vs Aβ*.

Activated Akt is not directly involved in the dephosphorylation of phosho-tau. In fact, activated Akt is capable of phosphorylating tau *in vitro* [34]. Several studies have shown that GSK-3β kinase inactivation by Akt-mediated phosphorylation of GSK-3β at Ser9 is involved in suppression of tau phosphorylation and protection of neurons [35]. Therefore, we investigated whether RNS60-mediated suppression of tau phosphorylation involves GSK-3β inactivation. Indeed, Aβ insult led to the suppression of GSK3β phosphorylation without altering the level of total GSK3β (Fig. 8A–B), and RNS60 pretreatment abrogated this effect (Fig. 8A–B). To determine whether GSK-3β activation is required for Aβ-induced tau phosphorylation, SHSY5Y cells were stimulated with Aβ for 3 h in the presence or absence of increasing concentrations of the GSK-3β inhibitor GSK-3βI. As observed by immunoblot analysis (Fig. 8C–D), GSK-3βI dose-dependently prevented Aβ-mediated increase in tau phosphorylation. At a dose of 10 µM, GSK-3βI almost completely blocked tau phosphorylation in response to Aβ challenge. These results implicate the involvement of GSK-3β in the observed tau phosphorylation and suggest that the effects of RNS60 are mediated through modulation of GSK-3β activity.

### RNS60 down-regulates GSK3β via the PI3K - Akt pathway

To further assess the relationship between the PI3K-Akt pathway and its down-stream target GSK3β in RNS60-mediated suppression of tau phosphorylation, SHSY5Y cells were treated with 1 µM Aβ for 30 min in the presence or absence of 2 µM LY and 10% RNS60. As evident from figure 8E-F, the PI3 kinase inhibitor LY294002 blocked the effect of RNS60 on GSK3β activation in SHSY5Y cells. Similarly, RNS60-mediated inactivation of GSK3β was abrogated by the Akt inhibitor Akti (Fig. 8G–H). Taken together, these findings suggest that RNS60 suppresses tau phosphorylation in neuronal cells, at least in part through the PI3K-Akt-GSK3β pathway (Fig. 8I).

### RNS60 treatment attenuates glial activation in the hippocampus of 5XFAD mice

**Figure 9:**
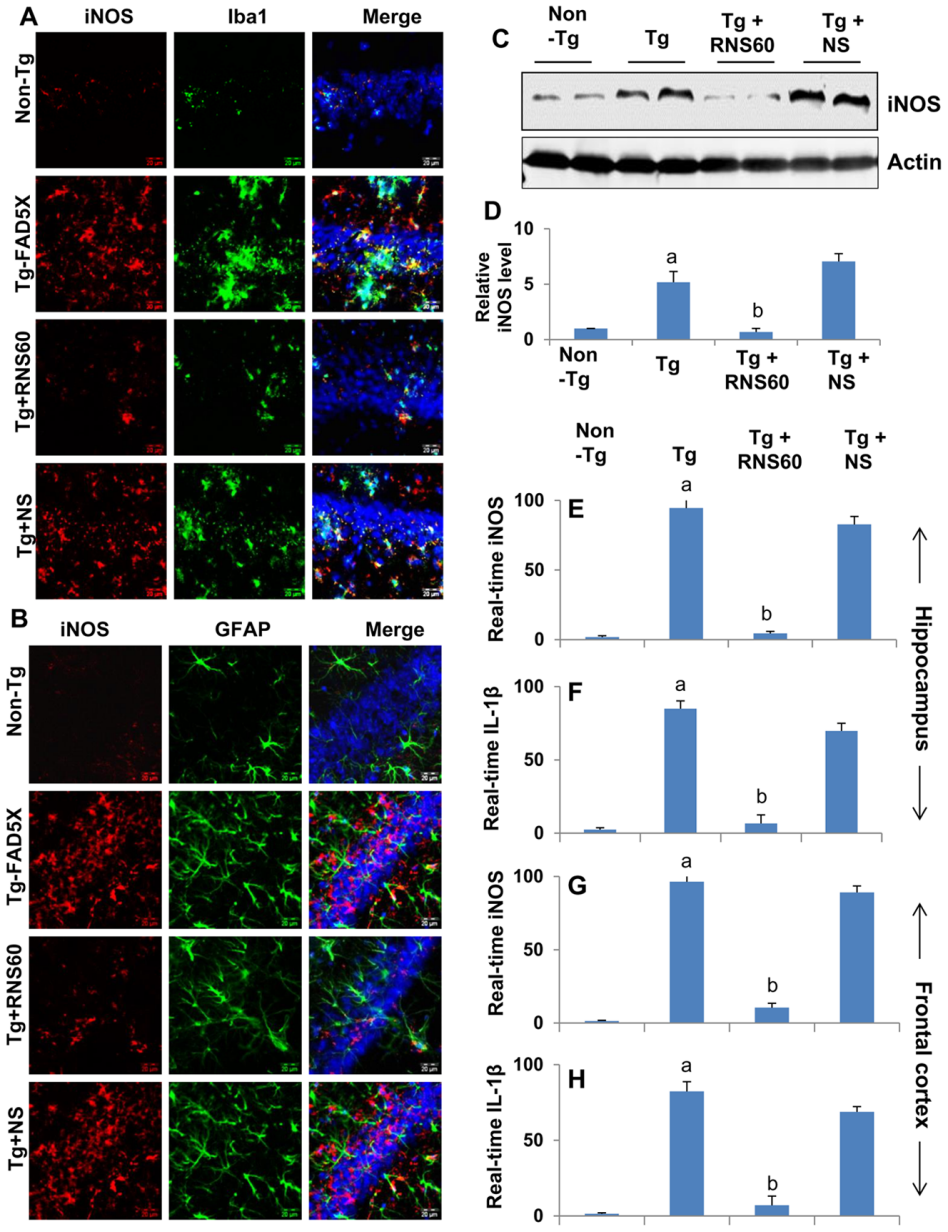
RNS60 treatment reduces glial activation in the hippocampus of Tg 5XFAD mice. Tg mice (5 months old) were treated with RNS60 and NS (300 µl/mouse/2d) via i.p. injection and after 2 months of treatment, hippocampal (CA1) sections were double-labeled for iNOS and either Iba1 (microglia) (A) or GFAP (astroglia) (B). The protein level of iNOS was analyzed in hippocampal homogenates by Western blot (C). Bands were scanned and results presented as iNOS/Actin (D). Results represent mean ± SEM of four mice per group. *^a^p<0.001 vs non-Tg; ^b^p<0.001 vs Tg*. The mRNA expression of iNOS (E & G) and IL-1β (F & H) was analyzed in hippocampal (E & F) and frontal cortex (G & H) samples by real-time PCR. Results represent mean ± SEM of four mice per group. *^a^p<0.001 vs non-Tg; ^b^p<0.001 vs Tg*.

Glial activation is becoming a hallmark of different neurodegenerative diseases including AD. Upon activation, glial cells express inducible nitric oxide synthase (iNOS) and generate nitric oxide to induce nitrosative stress [36]. Accordingly, the level of iNOS was much higher in the hippocampus of Tg mice than non-Tg mice (Fig. 9A–B). Double-label immunofluorescence analysis showed that this iNOS was expressed by both Iba1-expressing microglia (Fig. 9A) and astroglia (Fig. 9B). Next, we investigated if RNS60 treatment could suppress the level of iNOS in the hippocampus of Tg mice. As evident from immunofluorescence analysis, treatment of Tg mice with RNS60, but not NS, led to the inhibition of iNOS in the hippocampus (Fig. 9A–B). This is further supported by Western blot analysis of hippocampal homogenates for iNOS (Fig. 9C–D). Accordingly, RNS60 treatment also inhibited the mRNA expression of iNOS (Fig. 9E & G) and IL-1β (Fig. 9F & H) in hippocampus (Fig. 9E & F) and frontal cortex (Fig. 9G & H) of Tg mice. Together, these results indicate that RNS60 is capable of attenuating glial inflammation in vivo in the hippocampus of Tg mice.

### RNS60 treatment reduces plaque formation in the hippocampus of 5XFAD mice

**Figure 10:**
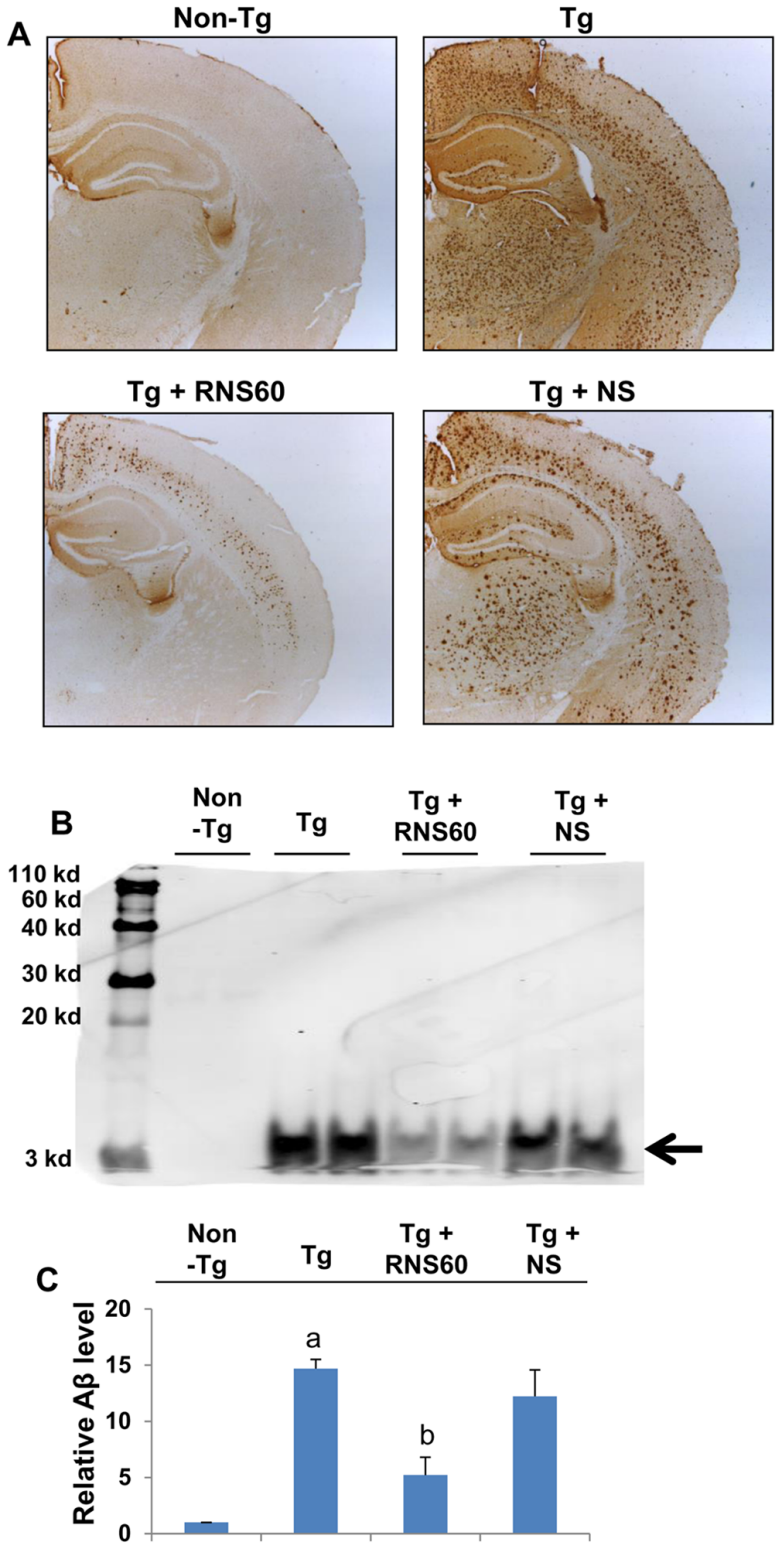
RNS60 treatment reduces the burden of amyloid beta from the hippocampus of Tg 5XFAD mice. Tg mice (5 months old) were treated with RNS60 and NS (300 µl/mouse/2d) via i.p. injection and after 2 months of treatment, hippocampal sections were immunolabeled with 82E1 antibody (A). Hippocampal homogenates were also analyzed for protein levels of Aβ by Western blot (B). Arrow indicates 4 kDa Aβ band. Bands were scanned and results presented as relative to control (non-Tg) (C). Results represent mean ± SEM of four mice per group. *^a^p<0.001 vs non-Tg; ^b^p<0.001 vs Tg*.

Aβ peptides are the main component of the amyloid plaques found in the brain of AD patients. Aβ is formed after sequential cleavage of the amyloid precursor protein (APP) by α-, β- and γ-secretases. The γ secretase that produces the C-terminal end of the Aβ peptide, cleaves within the transmembrane domain of APP, generating a number of isoforms of 36–43 amino acid residues in length [37]. The most common isoforms are Aβ40 and Aβ42, which are recognized by the 82E1 monoclonal antibody. We examined if RNS60 treatment was capable of reducing the load of Aβ in the hippocampus of 5XFAD mice. Immunostaining of hippocampal sections (Fig. 10A) as well as immunoblot analysis of hippocampal homogenates (Fig. 10B–C) with 82E1 antibody demonstrate that the level of Aβ peptides is markedly higher in the hippocampus of Tg mice as compared to non-Tg mice. Interestingly, treatment of Tg mice with RNS60, but not NS, led to significant decrease in Aβ (Fig. 10A–C), indicating that RNS60 is capable of reducing the burden of Aβ in the hippocampus of 5XFAD mice.

### RNS60 treatment protects spatial learning and memory in 5XFAD mice

**Figure 11:**
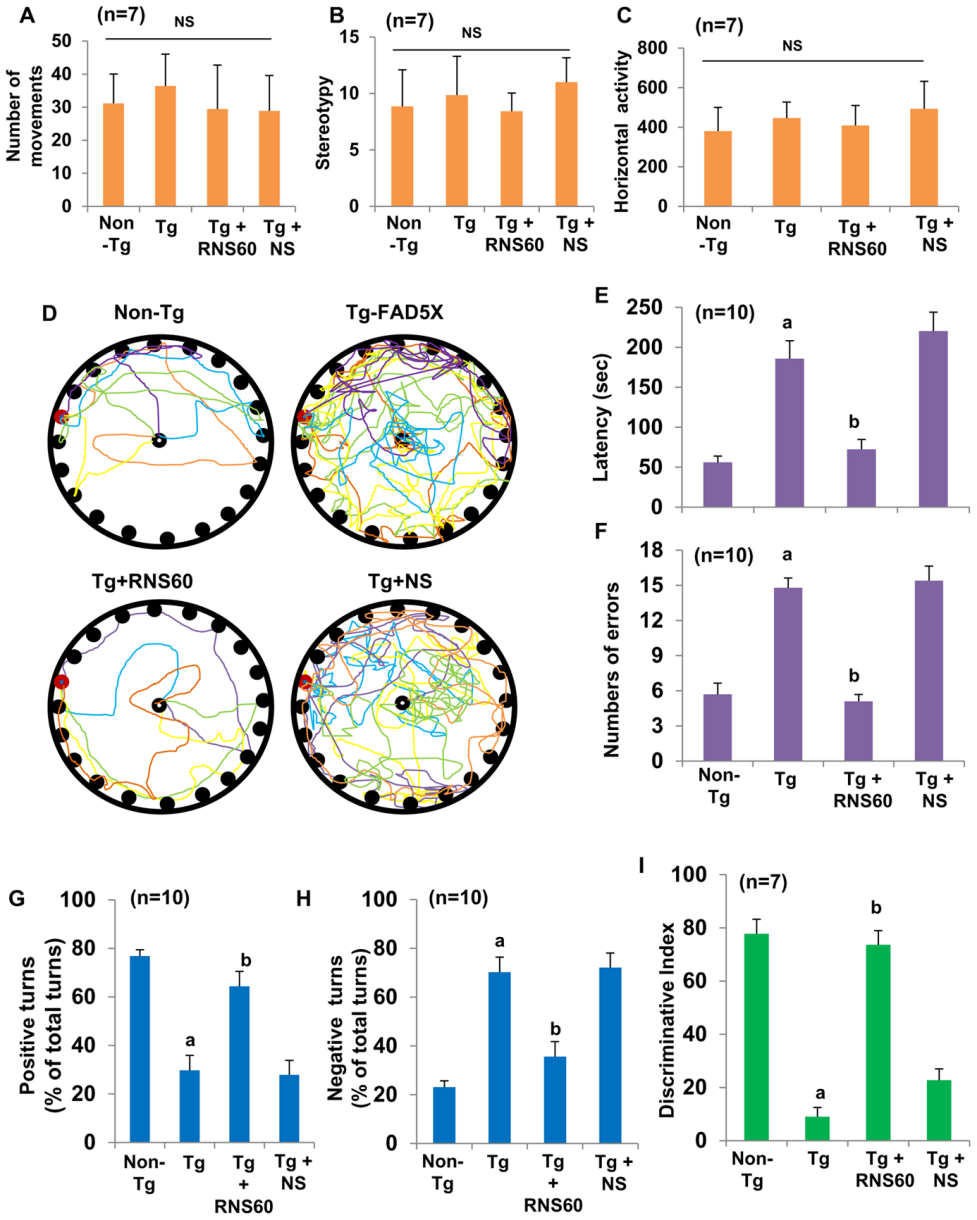
RNS60 treatment improves memory and learning in Tg 5XFAD mice. Tg mice (5 months old) were treated with RNS60 and NS (300 µl/mouse/2d) via i.p. injection and after 2 months of treatment, gross motor activities were examined by number of movements (*A*), number of stereotypy (*B*), horizontal activity (*C*) followed by monitoring spatial memory and learning by Barnes maze (track plots, D; latency, E; number of errors, F) and T maze (positive turns, G; negative turns, H). Short-term memory was also monitored by novel object recognition test, which is represented by discrimination index (I). While seven mice (n = 7) were analyzed for gross motor activities and short-term memory, ten mice (n = 10) were examined for Barnes maze and T maze. *^a^p<0.001 vs non-Tg; ^b^p<0.01 vs Tg*.

The ultimate therapeutic goal of neuroprotection in AD is to improve and/or protect memory. The hippocampus regulates the generation of long term memory and spatial learning. Therefore, to examine whether RNS60 protects only against structural damage or also against functional deficits seen in the 5XFAD model, we monitored Barnes maze and T maze activities. Barnes circular maze test, a hippocampus-dependent cognitive task, requires spatial reference memory. RNS60 did not significantly alter number of movements (Fig. 11A), stereotypy (Fig. 11B) and horizontal activity (Fig. 11C) in 5XFAD mice, suggesting that RNS60 does not modulate gross motor activities in this model. On the other hand, RNS60 significantly improved memory performance on Barnes maze as shown by latency [F_(3,36)_ = 20.90, p<0.000] and number of errors [F_(3,36)_ = 36.211 p<0.000]. *Post hoc* tests of multiple comparisons using Games-Howell analyses showed that 5XFAD mice did not find the hole easily (Fig. 11D) and exhibited more latency (p = 0.001, Fig. 11D) and higher errors (p<0.000, Fig. 11F) in Barnes maze as compared to non-Tg mice. However, RNS60-treated 5XFAD mice were as capable as healthy non-Tg in finding the target hole (Fig. 11D) and exhibited significantly less latency (p<0.003, Fig. 11E) and fewer errors (p<0.000, Fig. 8F) compared to untreated or NS treated 5XFAD mice.

Next, we performed T maze tests to determine whether RNS60 treatment improved spatial memory in 5XFAD mice. In this case as well, RNS60 treatment displayed significant effect on successful positive turns [F_(3,36)_ = 10.368, p<0.000] and number of errors [F_(3,36)_ = 22.777 p<0.000]. Untreated 5XFAD mice exhibited less number of positive turns (p<0.000) and more number of negative turns (p<0.000) than age-matched non-Tg mice in T maze apparatus (Fig. 11G–H). On the other hand, RNS60 significantly improved the hippocampus dependent memory performances as RNS60-treated 5XFAD mice exhibited higher number of positive turns (p<0.01) and less negative turns (p<0.01) compared to untreated or NS-treated Tg 5XFAD mice in T maze (Fig. 11G–H).

We also monitored short term memory by novel object recognition (NOR) task. The NOR task in particularly attractive as it requires no external motivation, reward, or punishment, and it can be completed in a relatively short time with minimal stress. We noticed markedly significant improvement F_(3,24)_ = 56.282, p<0.000 in short-term memory (Fig. 11I) as evidenced by discrimination index (i.e the difference between time spent exploring novel and familiar objects during test phase) in RNS60 treated 5XFAD mice as compared to untreated or NS-treated 5XFAD, but, 5XFAD mice showed profound impairment (p<0.000) as compared to age matched non-Tg mice.

## Discussion

Susceptibility to AD pathogenesis gradually increases with age, typically characterized by accumulation and oligomerization of Aβ1–42, resulting in the formation of amyloid plaques. The amyloid plaques eventually trigger a cascade of neurodegenerative events associated with inflammatory responses, tau hyperphosphorylation, synaptic dysfunction, neuronal death and ultimately clinical dementia. The development of safe and effective disease-modifying therapies that target the underlying pathophysiological process and alter the natural course of AD is a critical unmet need. Here we tested a saline-based agent, simple in its chemical composition, to achieve neuroprotection.report that treatment with RNS60, a saline-based agent devoid of an active pharmaceutical ingredient [15] can achieve neuroprotection. RNS60 is proposed to contain charge-stabilized nanostructures consisting of an oxygen nanobubble core surrounded by an electrical double-layer at the liquid/gas interface. In this study, we demonstrate that RNS60 is capable of suppressing fibrillar Aβ-induced neuronal apoptosis in human SHSY5Y neuronal cells. Neither RNS10.3 (saline processed without elevated oxygen) nor PNS60 (unprocessed saline with the same oxygen concentration as RNS60) had the same effect, demonstrating that the bioactivity of RNS60 is based neither on processing nor oxygen content alone.

Phosphatidylinositol 3-kinase (PI3-K) is a key signaling molecule implicated in the regulation of a broad array of biological responses including cell survival [38]. For class IA PI3-K, the p85 regulatory subunit acts as an interface by interacting with the IRS-1 through its SH2 domain and thus recruits the p110 catalytic subunit (p110α/β) to the cell membrane, which in turn activates downstream signaling molecules like Akt/protein kinase B and p70 ribosomal S6 kinase [38]. For class IB PI3-K, p110γ is activated by the engagement of G-protein coupled receptors. The p110γ then catalyzes the reaction to release phosphatidylinositol (3,4,5)-triphosphate as the second messenger, using phosphatidylinositol (4,5)-bisphosphate as the substrate, and activates downstream signaling molecules [39]. Therefore, we examined whether RNS60 activated PI3-K in neurons. We found that RNS60 induced the activation of p110β, but neither p110α nor p110γ, in primary human neurons, suggesting the specific activation of type IA p110β PI3-K in neurons. Recently, we have demonstrated that RNS60 induces the activation of type IA PI3-K in microglial cells [15]. However, in contrast to the specific activation of p110β PI3-K in neurons, RNS60 induced the activation of both p110α and p110β PI3-K in microglia [15]. Consistent with the fact that Akt is a downstream target of PI3-K [39], [40], we also observed increased phosphorylation of Akt in response to RNS60 treatment of human neurons and found that the protective effects of RNS60 require the activation of the PI3K-Akt pathway. The exact mechanism by which RNS60 induces activation of the p85α-associated p110β PI3-K signaling pathway in neurons is unclear at this time. In general, p85α-associated PI3-K is activated via growth factor receptors. Tyrosine phosphorylation of growth factor receptors creates docking sites for binding of p85α through its SH2 domains. Because RNS60 induces the activation of PI3K within minutes, it would not be surprising if RNS60 would employ growth factor receptors to activate type IA PI3K in neurons, either directly or indirectly via interacting with other accessory molecules.

The microtubule associated protein tau is a major component of neurofibrillary tangles and hyperphosphorylated tau is one of the most important neuropathological hallmarks of AD. Several studies have shown that Aβ toxicity is being mediated, at least in part, via increased phosphorylation of tau [10], [11], [41]. Studies have shown that cells challenged with Aβ exhibit increased levels of tau phosphorylation and that inhibition of tau phosphorylation via blocking of tau kinases can rescue cell death. Interestingly, neurons cultured from tau protein-knockout mice are resistant to Aβ toxicity [6]. Given the evidence that phosphorylated tau protein can lead to the destabilization of microtubules, impaired axonal transport and eventually neuronal death, it is generally believed that inhibition of tau phosphorylation induced by Aβ may be a useful therapeutic strategy for the treatment or prevention of AD. Here we demonstrate that RNS60 is capable of attenuating tau phosphorylation in Aβ-insulted cultured neurons and *in vivo* in the hippocampus of 5XFAD mice.

Increasing evidence has shed light on the role of GSK-3β in neurodegenerative diseases and on the fact that Aβ treatment results in increased GSK-3β activity in neuronal cells [11], [42]. Blockade of GSK-3β activity by lithium has been shown to reverse the Aβ-induced increase in tau phosphorylation and neurotoxicity in cultured neurons [43], [44]. GSK-3β activity can be regulated by multiple mechanisms, including the PI3K/Akt pathway; i.e., PI3K activates Akt by phosphorylation of Akt residue Ser473, which in turn phosphorylates GSK-3β at the Ser9 residue and thereby inhibits its activity. In the present study, RNS60 per se did not affect tau phosphorylation. This observation suggests that the preventive effect of RNS60 on the Aβ-induced increase in GSK-3β activity and tau phosphorylation is not due to a direct inhibition of the kinase and that additional signaling events are required. By employing inhibitors specific for PI3K, Akt, and GSK-3β, we were able to show conclusive evidence suggesting that RNS60 inhibits the activity of GSK-3β by stimulating the PI3K-Akt pathway.

Although glial activation has an important repairing function through scavenging of unwanted bodies in the CNS and recovery of injured CNS by actively monitoring and controlling the extracellular water, pH, and ion homeostasis, once microglia and astroglia become activated in the neurodegenerating microenvironment, activation always goes beyond control, and eventually detrimental effects of glial activation override its beneficial effects [27]. Activated glia produce NO, a number of proinflammatory cytokines, reactive oxygen species, etc., in excessive amounts for a prolonged time period that ultimately damage neurons and oligodendrocytes [36], [45]. Therefore, nowadays glial activation is considered to participate in the pathogenesis of several neurodegenerative disorders including AD. Recently we have observed that RNS60 suppresses the expression of proinflammatory molecules in glial cells via (type 1A PI3K-Akt-CREB)-mediated upregulation of IκBα [15]. Accordingly, here, we have also seen that RNS60 treatment is capable of suppressing glial activation *in vivo* in the hippocampus of 5XFAD mice.

While many drugs show therapeutic effect in cell culture models, very few exhibit efficacy *in vivo* in the brain. It is therefore remarkable that the data presented in this manuscript clearly establish that RNS60 is capable of suppressing AD-related pathological events in the hippocampus and protecting memory in 5XFAD mice. Treatment with RNS60 protected neurons from apoptosis, suppressed tau phosphorylation, reduced glial activation, and attenuated the burden of Aβ *in vivo* in the hippocampus, and most importantly ameliorated memory impairments in 5XFAD mice. We did not notice any drug related side effect (e.g. hair loss, weight loss, untoward infection etc.) in any of the mice used during the course of the study. These results suggest that RNS60 may be considered to mitigate neuronal death and protect memory in AD.

Although due to the unavailability of appropriate techniques for detecting nanobubbles, at present, we do not know whether and how RNS60 enters into the CNS, we have observed activation of type 1A PI-3 kinase, RNS60-specific signaling event, in the nigra of mice within only 3 h of intraperitoneal administration, suggesting that RNS60 may enter into the CNS [19]. Accordingly, peripheral administration of RNS60 protected dopaminergic neurons in the nigra of MPTP-intoxicated mice [19]. In another study, we have demonstrated that RNS60 treatment modulates both adaptive and innate immune responses and ameliorates the disease process of relapsing-remitting experimental allergic encephalomyelitis, an animal model of multiple sclerosis, in mice [46]. These results suggest that after peripheral administration, RNS60 is capable of exhibiting its effect in the brain.

Although experiments with SHSY5Y cells and mouse primary cortical neurons in culture and the 5XFAD mouse model certainly have limitations with respect to direct comparisons with the situation of neurons in the brain of AD patients, our results strongly suggest that RNS60 may be a novel tool to arrest Aβ-mediated neuronal apoptosis, glial activation and tau hyperphosphorylation, recognized pathological hallmarks seen in AD brains.
