# Correction: A Physically-Modified Saline Suppresses Neuronal Apoptosis, Attenuates Tau Phosphorylation and Protects Memory in an Animal Model of Alzheimer's Disease

**DOI:** 10.1371/journal.pone.0180602

**Published:** 2017-06-27

**Authors:** Khushbu K. Modi, Arundhati Jana, Supurna Ghosh, Richard Watson, Kalipada Pahan

In Fig 9B, the images in row Tg-FADX are incorrectly duplicates of the images in row Tg+NS, due to an error during preparation of the figure.

Here we provide a corrected Fig 9. The underlying images are provided as supporting information files. The authors confirm that these changes do not alter their findings.

**Fig 9:**
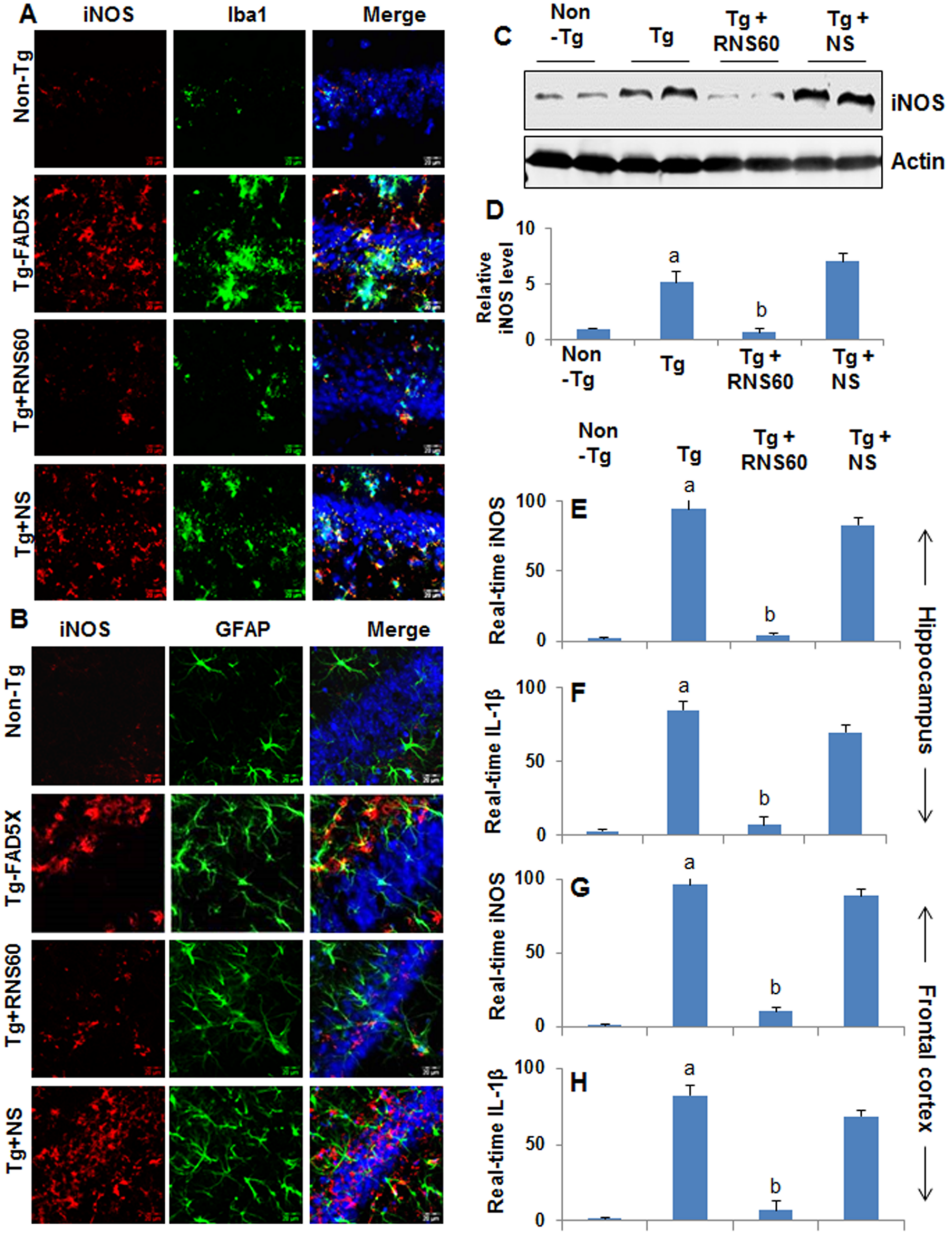
RNS60 treatment reduces glial activation in the hippocampus of Tg 5XFAD mice. Tg mice (5 months old) were treated with RNS60 and NS (300 μl/mouse/2d) via i.p. injection and after 2 months of treatment, hippocampal (CA1) sections were double-labeled for iNOS and either Iba1 (microglia) (A) or GFAP (astroglia) (B). The protein level of iNOS was analyzed in hippocampal homogenates by Western blot (C). Bands were scanned and results presented as iNOS/Actin (D). Results represent mean ± SEM of four mice per group. ^*a*^*p<0*.*001 vs non-Tg*; ^*b*^*p<0*.*001 vs Tg*. The mRNA expression of iNOS (E & G) and IL-1β (F & H) was analyzed in hippocampal (E & F) and frontal cortex (G & H) samples by real-time PCR. Results represent mean ± SEM of four mice per group. ^*a*^*p<0*.*001 vs non-Tg*; ^*b*^*p<0*.*001 vs Tg*.

## Supporting information

S1 File Underlying images for TG-FAD5X. (JPG)

S2 File Underlying images for TG+NS. (JPG)
