# Supplementary figures and images for: Correction: A Physically-Modified Saline Suppresses Neuronal Apoptosis, Attenuates Tau Phosphorylation and Protects Memory in an Animal Model of Alzheimer's Disease
Source: PLoS One. 2017 Jun 27;12(6):e0180602. doi: 10.1371/journal.pone.0180602 (PMC5487066; doi:10.1371/journal.pone.0180602)

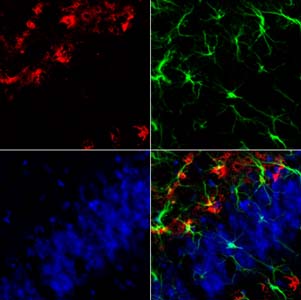

Supplement: S1 File — (JPG) [file pone.0180602.s001.jpg]

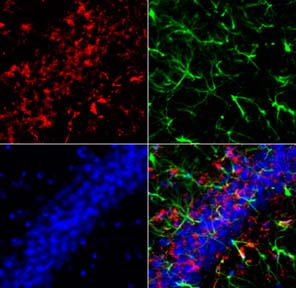

Supplement: S2 File — (JPG) [file pone.0180602.s002.jpg]
